# Substance Use among Adolescents Involved in Bullying: A Cross-Sectional Multilevel Study

**DOI:** 10.3389/fpsyg.2017.01056

**Published:** 2017-06-28

**Authors:** Jorge Gaete, Bernardita Tornero, Daniela Valenzuela, Cristian A. Rojas-Barahona, Christina Salmivalli, Eduardo Valenzuela, Ricardo Araya

**Affiliations:** ^1^Department of Epidemiology and Population Health, London School of Hygiene and Tropical MedicineLondon, United Kingdom; ^2^Department of Public Health and Epidemiology, Universidad de los AndesSantiago, Chile; ^3^Faculty of Education, Universidad de los AndesSantiago, Chile; ^4^School of Psychology, Universidad de los AndesSantiago, Chile; ^5^Faculty of Education, Pontificia Universidad Católica de ChileSantiago, Chile; ^6^Division of Psychology, University of TurkuTurku, Finland; ^7^Faculty of Social Sciences, Pontificia Universidad Católica de ChileSantiago, Chile; ^8^Centre for Global Mental Health and Primary Care Research, Institute of Psychiatry, Psychology and Neuroscience, King's College LondonLondon, United Kingdom

**Keywords:** bullying, substance use, adolescents, school-related factors

## Abstract

Being involved in bullying as a victim or perpetrator could have deleterious health consequences. Even though there is some evidence that bullies and victims of bullying have a higher risk for drug use, less is known about bystanders. The aim of this research was to study the association between bullying experience (as victims, bullies, or bystanders) and substance use. We gathered complete information from a nationally representative sample of 36,687 students (51.4% female) attending 756 schools in Chile. We used a self-reported questionnaire which was developed based on similar instruments used elsewhere. This questionnaire was piloted and presented to an expert panel for approval. We used multilevel multivariate logistic regression analyses, controlling for several variables at the individual (e.g., school membership, parental monitoring) and school levels (e.g., school type, school denomination). This study shows that bullies and bully-victims have a high risk for cigarette, alcohol, and cannabis use than bystanders. This is one of the few studies exploring the association between witnessing bullying and substance use. These findings add new insights to the study of the co-occurrence of bullying and substance use. Other factors, such as higher academic performance, stronger school membership, and better parental monitoring reduced the risk of any substance use, while the experience of domestic violence and the perception of social disorganization in the neighborhood, increased the risk. These findings may help the design of preventive interventions.

## Introduction

Aggressive behavior at school is a complex phenomenon, and bullying is probably the most common expression of it. Bullying is a major health problem in schools worldwide (National Academies of Sciences, Engeering, and Medicine, 2016). The most consensual definition of bullying includes the following elements (Farrington, 1993; Smith et al., 1999; Olweus, 2013; Menesini and Salmivalli, 2017): (i) Intentionality, i.e., the behavior has the intention to cause harm to another; (ii) Imbalance of power, i.e., a situation of unequal psychological or physical power, in favor of the bully; and (iii) Repetitiveness, i.e., a repeated action over time. Even though there is a general agreement in these elements of the definition, it is important to highlight some disagreements. For example, the Centers for Disease Control (CDC) explicitly excludes from the definition aggressive behaviors that occur between siblings, dating partners, or committed by adults, even if the acts take place in the school context.

The prevalence of bullying in schools varies from study to study, depending on the instruments used to measure it. Seminal research was conducted by Dan Olweus, who carried out an investigation including 750 representative schools (around 130,000 students) in Norway. This study showed that almost 15% of the students were involved in bullying (Olweus, 1993). Since then, many studies have been conducted, but mainly in high-income countries (Zych et al., 2015). A recent review across 80 studies found a mean prevalence rate of 35% for traditional bullying involvement and 15% for cyber bullying involvement (Modecki et al., 2014). In another large, cross-country comparison study, important differences were found between eastern and western countries (Sittichai and Smith, 2015), likely related to how students understand the meaning of bullying, and linguistic and cultural influences on its conceptualization (Menesini and Salmivalli, 2017). Overall, we can consider that between 10 and 30% of children and adolescents directly participate as bullies, victims, or bully-victims (Smith et al., 2002; Menesini and Salmivalli, 2017).

However, as was mentioned earlier, most bullying research has been conducted in developed countries (Zych et al., 2015), with more than three quarters of the most cited articles in bullying being published in North America and Northern Europe. Considering that some reports already show that violence and bullying are higher in less developed countries, especially in Latin America (Muula et al., 2009; Oliveros et al., 2009; Brito and Oliveira, 2013; Cassiani-Miranda et al., 2014; Lister et al., 2015), a future challenge within the field is to promote research in areas outside of North America and Northern Europe (Zych et al., 2015).

Bullying research is relatively new in Latin America (Wolf and Esteffan, 2008). In this context, the situation in Chile is not very different from the rest of the countries of the region. The first national study on school climate and school violence in Chile was conducted in 2005 (Ministerio De Salud, 2005). The results showed that 28% of students from 7 to 11th grades reported that they had been frequently insulted at school, compared with 9% who reported being frequently harmed. In addition, the results showed that such experiences were more prevalent in schools attended by students of lower socio-economic status, and more common among boys than girls. In 2009, the Chilean Government conducted another study to describe violent behaviors within school contexts. The results showed that 23.3% of students from the 7 to 12th grade reported having been a victim of aggressive behavior in schools and in 14.5%, this aggressiveness was repeated over time. Additionally, 28% of students admitted conducting an aggressive act toward others in school. Furthermore, this study reported an increase in more complex and severe aggressions, such us sexual abuse, weapon injuries, robberies, and thefts (Ministerio Del Interior, 2009). More recently, the Ministry of Education conducted a survey of 8th grade students in 5,855 schools. One out of five surveyed declared that harassment and threats were very frequent among school peers, 10% reported being victims of different types of aggression, and one fourth declared that they experienced this violence daily (Ministerio De Educacion, 2012).

There are typically at least three groups of students involved in bullying: bullies, victims, and bystanders (Salmivalli et al., 2011). Bullies are those students who play the leading role and commence the bullying. Victims are those who suffer the harassment from bullies. Some bullies may be also be victimized by other students; they are so-called bully-victims. Finally, bystanders are those students who witness the bullying. Studies have shown that ~85% of bullying episodes are witnessed by peers (Lynn Hawkins et al., 2001; Pepler et al., 2010). It is important to note that within each of these main groups, there are different types of students (National Academies of Sciences, Engeering, and Medicine, 2016).

Several factors associated with bullying have been reviewed by other authors (Olweus, 2013; National Academies of Sciences, Engeering, and Medicine, 2016; Menesini and Salmivalli, 2017). For the purposes of this study, we will focus on studies exploring the association between bullying experience and substance use.

Several studies have found an association between bullying behaviors and substance use among adolescents (Kaltiala-Heino et al., 2000; Haynie et al., 2001; Morris et al., 2006; Liang et al., 2007; Carlyle and Steinman, 2007; Wolf and Esteffan, 2008; Tharp-Taylor et al., 2009; Luk et al., 2010, 2012; Centers for Disease Control Prevention, 2011; Vieno et al., 2011; Radliff et al., 2012; Gámez-Guadix et al., 2013; Espelage et al., 2014). Research has found that there is a strong association between tobacco use and bullies or bully-victims (Morris et al., 2006; Vieno et al., 2011), or victims (Tharp-Taylor et al., 2009). In a similar manner, alcohol use is strongly associated with those students having aggressive behaviors, such as bullies and bully-victims (Kaltiala-Heino et al., 2000; Vieno et al., 2011), and other studies have found that being a victim is an important risk factor for alcohol consumption (Sullivan et al., 2006; Tharp-Taylor et al., 2009; Luk et al., 2010; Vieno et al., 2011; Radliff et al., 2012). Finally, cannabis use is strongly associated with being a bully or a bully-victim (Radliff et al., 2012). Moreover, longitudinal studies have also shown the association between bullying and substance use. For instance, self-reported bullying at Grade 5 (when students are 11 years-old) predicts violent behavior and problematic consumption of alcohol and drugs 6 years later (Kim et al., 2011), with similar results reported in another study (Niemelä et al., 2011). However, there is less information about these associations and participation as bystander in the bullying experience (Rivers et al., 2009; Durand et al., 2013).

Considering the hierarchical structure of school-based data (students being nested within classes nested within schools) multilevel modeling is the most appropriate approach to analyze it. However, few studies have explored these associations using multi-level modeling. For instance, Bradshaw et al. (2013) developed three-level hierarchical linear models with the purpose of exploring the association between the different roles involved in bullying behavior (victim, bully, bully-victim, and no involvement) and three health-risk behaviors: violence, substance use (alcohol, cigarette, cannabis, and prescription drug), and academic problems. In general, the results showed that all students involved in bullying behavior were more likely to consume different kinds of substances. The group at greatest risk consisted of bully-victims, followed by bullies and victims, respectively.

Much less information can be found in Latin American countries (Rodríguez et al., 2006; Andrade et al., 2012; Córdova Alcaráz et al., 2012; Cassiani-Miranda et al., 2014). In the 2009 National School Health Survey in Brazil, results showed that both boys and girls who were bullies or victims were at a heightened risk of consuming illegal drugs and alcohol (Andrade et al., 2012). Similarly, in Peru, the Second National Study of Drug Consumption and Prevention reported that being a victim of bullying was associated with the abuse of legal, illegal, and medical drugs (Romaní and Gutiérrez, 2010).

The explanation of this association needs further investigation. Research has shown that bullies may be involved in drug use as part of a more general involvement in antisocial behavior, or as a byproduct of their engagement with groups of peers presenting deviant behaviors (Ttofi and Farrington, 2011). Fighting or verbal and relational aggression, seem to be part of a cluster of *problem behaviors*, insofar as substance use and bullying show reciprocal relations (Espelage et al., 2014). Several studies show that this association is stronger among bullies than victims (Kaltiala-Heino et al., 2000; Nansel et al., 2001; Carlyle and Steinman, 2007; Radliff et al., 2012; Gámez-Guadix et al., 2013). For instance, data from the 2009 Massachusetts Youth Health Survey suggests that middle school students who are bullies or bully-victims are three to six times more likely to admit substance use than their victims or those not involved in bullying (Centers for Disease Control Prevention, 2011). It is possible that victims may use substances as a coping mechanism to deal with the anxiety produced by the attacks and rejection by their peers (Carlyle and Steinman, 2007; Continente et al., 2010; Romaní and Gutiérrez, 2010). Even though, much less information is available exploring the association between being a bystander (with no direct involvement in bullying actions) and substance use (Rivers et al., 2009), it is possible to hypothesize that these students may have a higher risk of substance use when compared to students with no involvement. A recent study that explored the effect of adolescent exposure to violence (witnessing or awareness of) on subsequent adult illicit drug use found that witnessing parental violence and exposure to neighborhood violence during adolescence was significantly associated with adult drug use (Menard et al., 2015). This study also suggested that the exposure to violence in adolescence is especially hazardous because this is the period of life when illicit drug use is typically initiated (Elliott et al., 1989). In support of this, the general strain theory (Agnew, 1985) proposes that strains are present when individuals are exposed to noxious stimuli, which may include witnessing of violence. This strain may lead to different modes of adaptation, such as engagement in deviant behavior or delinquency (Agnew, 1985; Broidy, 2001), or using drugs as a form of self-medication (Harrison et al., 1997). Several studies have shown that children and adolescents exposed to violence often experience anxiety and social concerns (Groves, 1999; Kitzmann et al., 2003; Meltzer et al., 2009), which in turn may lead them to use drugs to reduce those symptoms.

Finally, substance use in Chile is an important public health problem. In the last national study surveying students from 8 to 12th grade, 22.9% reported any alcohol consumption in the last month and 8.4% reported tobacco use every day (SENDA, 2015). In relation to cannabis, cocaine, and cocaine base (*Pasta base*), the monthly prevalence in 2015 was found to be 13.5, 1.6, and 0.3%, respectively. Additionally, there has been an increase in the prevalence reported since the previous survey (2012) at least for alcohol and cannabis use (SENDA, 2015). Therefore, exploring the factors associated to substance misuse, will provide valuable information in the implementation of preventive measures, as there is already some evidence showing that anti-bullying preventive programs may also reduce substance use among adolescents (Olweus, 1993; Durand et al., 2013). Several individual, family, school, peer, and community factors have been associated with substance use among adolescents (Tyas and Pederson, 1998; Monasterio, 2014). For example, students with behavioral problems have a higher risk for drinking and cannabis use, and peer smoking and drinking have also been associated with the use of multiple drugs (Harakeh et al., 2012; Tomczyk et al., 2015). Additionally, the children of parents who provide support and clear limits and norms are less likely to drink (Berge et al., 2016). Similarly, poor parental monitoring is associated with tobacco, alcohol and cannabis use (Gaete and Araya, 2017). Regarding school factors, poor school bonding increases the likelihood of substance use (Gaete and Araya, 2017), and strong anti-tobacco polices at school have been associated with less smoking (Wiium et al., 2011; Galan et al., 2012; Paek et al., 2013). Therefore, adjusting for these variables in the analyses of the association between bullying experience and substance use seems a reasonable approach.”

Therefore, this study aimed to explore the association between different degrees of bullying involvement and alcohol, tobacco, and cannabis use at the individual level, taking into account personal (e.g., school membership), family (parental monitoring), and community factors (perception of violence in the neighborhood) at the individual level, and contextual factors (e.g., school type) at the school level. To our knowledge, this is the first multilevel study in Latin-America exploring this association, and this is one of the few studies exploring these associations in the different groups that play a role in bullying. Specifically, individuals with no experience of bullying, bystander only, victim only, bully only, and bully-victim. Our general hypothesis is that the exposure to bullying in adolescence, as perpetrator, victims or as bystander, will increase the risk for substance use.

## Materials and methods

### Study design

This is an analytical cross-sectional study that uses a self-reported survey.

### Setting, sample

Data came from the Forth National Survey of Violence in School Context 2014 (ENVAE 2014) in Chile. This survey used a stratified nationally representative sample of students, attending 7 to 12th grades, in state, subsidized, and private schools in urban municipalities of Chile. This study used simple random sampling, stratified in two stages (schools and students), with a probability proportional to the number of students in each school.

The sample frame for this study was 944 schools. It was planned to randomly invite 60 students per school to participate in the survey. All schools were contacted prior to data collection to obtain permission and send consent documents to parents or caregivers. A total of 756 schools agreed to participate, reaching out 45,360 parents and caregivers. Around of 84% of students were given consent and attended the day of the survey. Data collection took place during one school day, on a date pre-determined by the school authorities, between September and December 2014. No replacements were implemented. Finally, complete data from 37,931 (51.4%, females) students from 754 schools was collected.

This secondary analysis study was approved by the Bioethical Committee of Universidad de los Andes (Chile) (August 9th, 2016). It was performed in accordance with the Declaration of Helsinki. All participants provided informed consent.

### Measures

#### School questionnaire

The self-report questionnaire consisted of questions regarding students' demographic features, questions regarding school violence (including bullying experience), academic performance, school norms awareness, school bonding, school membership, perception of peer substance use at school, parental monitoring, family maltreatment, violence and drug-related actions in the neighborhood, and substance use. The expert committee responsible for the study built most of the items and scales. A pilot study (*n* = 117) was conducted during July 2014 to assess the structure and length, wording, and understanding of the questionnaire. The expert panel approved the final version of the questionnaire.

#### Dependent variables

We used a frequency measure of substance use, asking students how many days during the last 30 days prior to the study they had used tobacco or alcohol (e.g., “How many times in the last 30 days have you smoked cigarettes?”; “How many times in the last 30 days have you used alcohol?” The answer ranged from 0 = Never to 5 = More than 40 times), and how many days during the last 12 months prior to the study they had used cannabis (e.g., “How many times in the last 12 months have you used or smoked cannabis? The answer ranged from 0 = Never to 5 = More than 40 times). Then, we categorized the answers into two possibilities: 0 = Not user and 1 = User (smoking cigarette or drinking in any of the previous 30 days; or using cannabis in any of occasion of the previous 12 months). The 30-day period for tobacco and alcohol use, and the 12-month period for cannabis use are standard and recommended time intervals used in school surveys to define current users (United Nations Office on Drugs and Crime (UNODC), 2003), and the binary approach is widely utilized, allowing us to compare our results with other studies (Hibell et al., 2012; Johnston et al., 2016).

#### Independent variables

##### At individual-level

A structured questionnaire covering socio-demographic factors, bullying experiences, other school-related factors, and family and community factors was administered to each student.

*Socio-demographic features*. Information about age, sex, and school year was gathered.

*Bullying experience*. Three independent scales, using a similar set of 9 different situations that students may have been experienced in the current academic year, were used to identify students who had been victims of bullying, bullies, or bystanders (observing bullying actions with no direct involvement). The nine items of the scale for victims were: During the current academic year, how often have other students in your school (a) spread lies or hurtful things about you; (b) hit or kicked you; (c) threatened you; (d) thrown heavy objects at you with the intention of hurting you; (e) ignored or excluded you; (f) insulted you; (g) said mean names to you; and (h) pushed you with the intention of hurting you. Students answered to each event using a 5-point scale (1 = never; 2 = 2 or three times during the current academic year; 3 = every month; 4 = every week; 5 = every school day). The scales exploring bullying perpetration and witnessing of bullying (bystanders) used the same set of 9 items, but the wording was changed accordingly. We re-arranged these answers and considered a bullying experience if the students responded having experienced any of these actions at least every month. Therefore, three new bullying binary variables were created for each scale: Being a victim (0 = No, 1 = Yes); Being a bully (0 = No, 1 = Yes); and Being a bystander (0 = No, 1 = Yes). Later, we combined these three variables into one new variable were students could be identified in mutually exclusive categories: 0 = having no experience of bullying actions (If the student responded “No” to all three bullying binary variables), 1 = being a bystander only (If the student responded “Yes” to Being a bystanders, but “No” to Being a victim and Being a bully); 2 = being a victim only (If the student responded “Yes” to Being a victim, but “No” to Being a bystander and Being a bully), 3 = being a bully only (If the student responded “Yes” to Being a bully, but “No” to Being a victim and Being a bystander); and 4 = being a bully-victim (If the student responded “Yes” to Being a victim and Being a bully, but “No” to Being a bystander).

##### Other school-related factors

*Self-reported academic performance*. In Chile, the grade point average [GPA] scale goes from 1 to 7, where 7 is the highest GPA, possible answers were arranged in six categories from 1 = < 4.5 to 6 = 6.5–7.0). The higher the score, the better the academic achievement.

*School norms awareness*. This is a 6-item Likert scale (1 = strongly disagree to 5 = strongly agree). Items explore several aspects of awareness of school's norms (for example, “I know the school coexistence norms,” “the school coexistence norms are known by my parents”). The higher the score, the higher the awareness of school's norms. This is a unidimensional scale, with internal reliability, alpha = 0.78.

*School bonding*. One item explores this variable asking “generally, how happy are you to go to school?” with 5 potential answers (1 = not at all happy to 5 = extremely happy). A higher score represents a better school bonding. This item was taken from a questionnaire used in the World Health Organization cross-national study called “Health Behavior in School-Aged Children” (HBSC) (Currie et al., 2002).

*School membership*. This is a 15-item scale exploring the sense of belonging at the school (e.g., “I feel proud of my school,” “I feel part of this school,” and “I would like to be in this school next year”), if the students feel respected by the teachers and/or school staff (“Teachers in this school respect me,” “The school authorities listen to students' ideas”), and if there are good relationships between students and teachers (“There is trust in the relationship between students and teachers”). Students responded how much they agreed with each statement (1 = strongly disagree to 5 = strongly agree). A higher score means having a higher sense of school membership. This is a unidimensional scale, with internal reliability, alpha = 0.90.

*Perception of peer substance use at school*. This is a 4-item scale exploring the perception of peer substance use at school (cigarettes, alcohol, and other drugs). For example, students were asked: How often have you observed other students smoking cigarettes at your school? Responses ranged from 1 = never to 5 = every day. A higher score represents students perceiving a high frequency of other students using drugs at school. This is a unidimensional scale, with internal reliability, alpha = 0.83.

##### Family factors

*Parental monitoring*. This is a 7-item scale exploring how much or how frequently parents are aware or know about students' activities in the school, with friends, and after school. Responses ranged from 1 = little/never to 4/5 = very much/always. A higher score means that parents are well aware of where and with whom the students are spending time (i.e., better parental monitoring; this is a unidimensional scale, with internal reliability, alpha = 0.85).

*Maltreatment*. This is a 6-item scale exploring maltreatment experience at home. For example, the students were asked how often during the last year, they have experienced insults or threats from parents or any other adult at home. Answers ranged from 1 = never to 5 = every day. A higher score means higher frequencies of maltreatment experiences at home. This is a unidimensional scale, with internal reliability, alpha = 0.81.

##### Community disorganization

We asked students if they have perceived or experienced some of the following actions in their neighborhood, specifically (1) People selling drugs; (2) Fights in the streets; (3) People using drugs in the streets; (4) Assaults; (5) People carrying guns; (6) Bullet sounds; and (7) Unsafe places for walking. Responses for each item range from 1 = never to 5 = every day. This variable may be considered as related to the social disorganization theory (Shaw and Mckay, 1972), which considers that unsafe communities may increase violence and substance use among those who live there (Rutter et al., 2011). A higher score means a higher level of community disorganization. This is a unidimensional scale, with internal reliability, alpha = 0.90.

##### At school-level

The following information was collected from schools: type of school (municipal state-funded, subsidized, private), school co-educational status (only boys, only girls, mix gender), number of teachers per school (1 = small [<20 teachers at the school]; 2 = medium [between 20 and 49 teachers at the school]; 3 = large [≥50 teachers at the school]), school denomination (1 = secular; 2 = Catholic; 3 = religious non-Catholic), and co-educational status (1 = only boys; 2 = only girls; 3 = mixed).

### Data analyses

Complete data analyses, using listwise deletion, were performed. General descriptive statistics were used to characterize the sample. The association between variables was assessed using multilevel logistic regression models for each substance. Multilevel analysis is recommended when data comes from hierarchical levels. In this study, the students belonged to schools where they share context; therefore, we expected the same degree of similarity between their behaviors. In other words, observations are not completely independent of one another (Delprato, 1999; Khan and Shaw, 2011). Multilevel logistic regressions allows for examination of effects at the individual- and school-levels, as well as any other contextual factors of student behaviors (Hox, 2002; Rabe-Hesketh and Skrondal, 2005). Therefore, we defined two levels, individual and school levels.

Different models were built. The null model was the reference and gave evidence of the existence of substance use variation between schools. Model 1 included the univariable analysis for each variable. Those variables associated at a significance level of *p* < 0.05 were included in Models 2 and 3. Model 2 included all individual-level variables. Model 3 included all school-level variables. The final full model included all individual- and school-level variables that were associated with each dependent variable in Models 2 and 3, at a significance level of *p*-value < 0.05. Sex and age were included in all full models, regardless of the strength of the association reached in other models, because they are considered important confounding variables. The fit for all full models was assessed using the C-statistics, along with the 95%CI, where a C-statistic of 1 is a perfect fit model and 0.5 is no better than chance (Hosmer and Lemeshow, 2000). A good fit model should have a C-statistic >0.7 (Hosmer and Lemeshow, 2000). Stata 14.1 was used for all analyses.

## Results

### Description of participants

Complete data from 37,931 (51.4%, females) students from 754 schools was collected. The mean age was 14.9 (SD = 1.8) years old. Students were attending grades 7–12 (see Table 1). For data regarding bullying experience, 36,687 students provided complete data. As such, all following results are based upon the completed data of these students (see Tables 1–3).

**Table 1:** Description of individual level variables.

**Student variables**	***n***	**% (95%CI)/Mean (*SD*)**	**Skewness**	**Kurtosis**
**PERSONAL DOMAIN**				
Age	37,931	14.9 (1.8)		
Gender (Female)	37,931	51.4 (50.0–53.0)		
**SCHOOL GRADE LEVEL**				
Year 7	7,843	20.6 (19.5–22.1)		
Year 8	7,732	20.4 (19.0–21.8)		
Year 9	6,425	16.9 (16.1–17.8)		
Year 10	6,188	16.2 (15.3–17.1)		
Year 11	5,900	15.3 (14.5–16.2)		
Year 12	4,147	10.6 (9.8–11.4)		
**BULLYING EXPERIENCE**				
Victim only	7,723	21.2 (20.6–21.8)		
Bully only	1,960	5.3 (5.0–5.6)		
Bully-victim	4,196	11.5 (11.0–12.1)		
Bystanders only	17,135	47.3 (46.5–48.1)		
**SUBSTANCE USE**				
Cigarette	7,589	21.0 (20.2–21.8)		
Alcohol	9,094	24.7 (23.9–25.6)		
Cannabis	5,445	14.7 (14.0–15.5)		
**SCHOOL DOMAIN**				
Self-Reported Academic Performance				
<4.5	750	2.0 (1.8–2.3)		
4.5–4.9	4,155	11.0 (10.4–11.7)		
5.0–5.4	9,400	25.0 (24.2–25.8)		
5.5–5.9	12,506	33.4 (32.7–34.1)		
6.0–6.4	7,988	21.2 (20.4–22.1)		
6.5–7.0	2,741	7.3 (6.8–7.9)		
School policy awareness	36,979	3.6 (0.7)	−0.60	3.66
School bonding	37,957	3.2 (1.1)	−0.25	2.69
School membership	34,694	3.7 (0.6)	−0.51	3.53
Perception of peer drug use in school	37,339	1.6 (0.9)	1.84	5.80
**FAMILY DOMAIN**				
Parental monitoring	35,673	25.9 (5.5)	−1.25	4.23
Maltreatment	36,454	1.4 (0.6)	2.62	11.94
**COMMUNITY DOMAIN**				
Community disorganization	36.091	1.6 (0.9)	1.81	5.70

*CI, Confidence Interval; SD, Standard Deviation*.

**Table 2:** Description of school level variables.

**School variables**	***n***	**% (95%CI)**
**SCHOOL TYPE**		
Municipal state-funded	16,170	40.9 (37.0–44.8)
Subsidized	20,473	54.4 (50.5–58.3)
Private	1,643	4.7 (3.3–6.8)
**NUMBER OF TEACHERS PER SCHOOL**		
Small (<20 teacher per school)	12,647	33.0 (29.4–36.9)
Medium (between 20 and 49)	22,574	58.9 (54.9–62.7)
Large (≥50)	3,065	8.1 (6.2–10.5)
**SCHOOL DENOMINATION**		
Secular	24,799	62.1 (60.1–67.8)
Catholic	10,938	29.8 (26.2–33.6)
Religious, non-Catholic	2,362	6.2 (4.5–8.4)
**SCHOOL CO-EDUCATIONAL STATUS**		
Only boys	934	2.3 (1.4–3.8)
Only girls	1,989	6.0 (4.3–8.3)
Mixed	35,176	92.0 (89.1–93.6)

*CI, Confidence Interval; SD, Standard Deviation*.

**Table 3:** Individual level variables according to bullying experience sub-samples.

**Student variables *N* total = 36,687**	**No experience of bullying *n* = 5,652**	**Bystanders *n* = 17,156**	**Victim *n* = 7,723**	**Bully *n* = 1,960**	**Bully-victim *n* = 4,196**
		**% (95 CI)/Mean (*SD*)**	**% (95 CI)/Mean (*SD*)**	**% (95 CI)/Mean (*SD*)**	**% (95 CI)/Mean (*SD*)**
**PERSONAL DOMAIN**					
**Age**					
Gender (Female)					
Male	47.0 (44.8–49.1)	45.4 (43.8–47.0)	48.5 (46.7–50.4)	56.3 (53.3–59.3)	58.9 (56.5–61.2)
Female	53.0 (50.9–55.2)	54.6 (53.0–56.2)	51.4 (49.6–53.3)	43.7 (40.7–46.7)	41.1 (38.8–43.5)
**Grade Level**					
Year 7	21.4 (19.4–23.6)	17.2 (15.9–18.6)	25.2 (23.4–27.0)	19.4 (17.0–21.9)	24.7 (22.5–27.0)
Year 8	20.5 (18.6–22.6)	18.3 (17.0–19.8)	23.3 (21.6–25.1)	19.9 (17.6–22.3)	23.7 (21.6–26.0)
Year 9	17.5 (16.0–19.2)	17.4 (16.5–18.4)	15.7 (14.4–17.0)	17.1 (15.1–19.3)	15.8 (14.3–17.5)
Year 10	14.4 (13.0–16.0)	17.2 (16.2–18.2)	15.4 (14.1–16.7)	17.5 (15.5–19.7)	16.0 (14.5–17.7)
Year 11	16.2 (14.6–17.8)	17.8 (16.8–18.8)	11.7 (10.7–12.8)	15.3 (13.5–17.4)	11.2 (9.9–12.6)
Year 12	10.0 (8.8–11.3)	12.1 (11.2–13.1)	8.8 (7.8–9.9)	10.8 (9.2–12.6)	8.6 (7.4–10.0)
**SUBSTANCE USE**					
Cigarette	14.2 (13.0–15.4)	20.0 (19.0–20.9)	21.7 (20.4–23.0)	30.0 (27.6–32.4)	29.6 (27.8–31.4)
Alcohol	16.7 (15.4–18.1)	24.4 (23.3–25.4)	24.6 (23.3–26.0)	36.3 (33.8–38.8)	33.5 (31.7–35.4)
Cannabis	8.3 (7.4–9.2)	14.0 (13.2–14.8)	14.7 (13.6–15.8)	24.6 (22.2–27.0)	22.8 (21.1–24.5)
**SCHOOL DOMAIN**					
Self-Reported Academic Performance					
<4.5	1.5 (1.2–2.0)	1.8 (1.5–2.1)	2.0 (1.6–2.4)	3.5 (2.6–4.6)	2.8 (2.2–3.5)
4.5–4.9	9.9 (8.9–11.0)	10.1 (9.4–10.9)	12.1 (11.1–13.1)	13.4 (11.7–15.3)	12.2 (10.9–13.6)
5.0–5.4	25.1 (23.5–26.7)	23.7 (22.7–24.7)	26.7 (25.4–28.0)	26.7 (24.5–29.0)	26.2 (24.5–27.9)
5.5–5.9	34.6 (33.1–36.1)	33.1 (32.3–34.0)	32.9 (31.7–34.1)	31.6 (29.5–33.8)	34.3 (32.6–36.0)
6.0–6.4	21.6 (20.3–23.1)	22.8 (21.8–23.80)	19.7 (18.6–20.9)	19.1 (17.2–21.3)	18.8 (17.4–20.4)
6.5–7.0	7.2 (6.4–8.2)	8.4 (7.7–9.2)	6.6 (5.9–7.3)	5.7 (4.6–7.0)	5.7 (4.8–6.7)
School policy awareness	3.9 (0.7)	3.6 (0.7)	3.5 (0.7)	3.5 (0.7)	3.4 (0.7)
School bonding	3.6 (1.0)	3.3 (1.0)	3.1 (1.1)	3.1 (1.1)	3.0 (1.1)
School membership	4.0 (0.6)	3.7 (0.6)	3.6 (0.7)	3.6 (0.6)	3.5 (0.7)
Perception of peer drug use in school	1.2 (0.4)	1.5 (0.8)	1.8 (1.0)	1.8 (1.0)	1.9 (1.1)
**FAMILY DOMAIN**					
Parental monitoring	26.7 (5.6)	262 (5.1)	25.5 (5.8)	24.5 (5.6)	24.1 (5.8)
Maltreatment	1.2 (0.3)	1.3 (0.5)	1.5 (0.6)	1.6 (0.7)	1.7 (0.8)
**COMMUNITY DOMAIN**					
Community disorganization	1.3 (0.7)	1.6 (0.9)	1.8 (1.0)	1.9 (1.1)	2.0 (1.1)

*CI, Confidence Interval; SD, Standard Deviation*.

### Characterization of students according to their bullying experience

A total of 21.1% of students reported being a victim of bullying, 5.3% identified themselves as a bully and 11.4% reported being a bully-victim. Additionally, 46.8% of the students reported being bystanders of bullying situations in their schools. Finally, 15.4% of the sample reported that they had not experienced or perceived any action of bullying at their schools.

In the personal domain, being a victim or a bystander was more frequent among female students, while being a bully or a bully-victim was most common among boys. Any bullying experiences were most frequent among 7 and 8th graders, especially victimization and bullying others.

Bullies were the students who reported more substance use (cigarettes, 30%; alcohol, 36.3%; cannabis, 24.6%). Conversely, students who were not exposed to any experience of bullying had the lowest substance use rates.

Regarding the school domain, bully-victims showed the lowest school policy awareness, school bonding, school membership, and perceived the most of peer drug use at school.

Additionally, bully-victims reported low level of parental monitoring, and high levels of maltreatment and community disorganization (see Table 3).

### Cigarette smoking and associated factors

The full model had a good fit (C-statistic = 0.77). Regarding bullying experience, students who had any kind of bullying experience, compared with students with no bullying experience, had a higher risk for smoking, especially among those who bullied others. The risks were similar between victims (OR = 1.14; 95%CI: 1.00–1.29) and bystanders (OR = 1.16; 95%CI: 1.04–1.29), and between bullies (OR = 1.59; 95%CI: 1.34–1.89) and bully-victims (OR = 1.60; 95%CI: 1.40–1.83) (see Table 4).

**Table 4:** Multilevel logistic regression analysis: cigarette smoking.

	**Cigarette Smoking**				
	**Model 0**	**Model 1**	**Model 2**	**Model 3**	**Full model**
		**OR (95%CI)**	**OR (95%CI)**	**OR (95%CI)**	**OR (95%CI)**
**INDIVIDUAL LEVEL**					
**Personal Domain**					
Age		1.37 (1.35–1.40)	1.34 (1.31–1.37)		**1.35** (1.32–1.37)
Gender (ref. Male)		1.22 (1.15–1.29)	1.51 (1.40–1.62)		**1.50** (1.40–1.61)
**Bullying Experience**					
No experience		1	1		1
Bystander only		1.43 (1.31–1.56)	1.15 (1.04–1.28)		**1.16** (1.04–1.29)
Victim only		1.60 (1.45–1.77)	1.13 (1.00–1.28)		**1.14** (1.00–1.29)
Bully only		2.56 (2.22–2.94)	1.57 (1.32–1.87)		**1.59** (1.34–1.89)
Bully-victim only		2.64 (2.37–2.93)	1.59 (1.39–1.82)		**1.60** (1.40–1.83)
**School Domain**					
Self-Reported Academic Performance		0.68 (0.66–0.70)	0.75 (0.72–0.77)		**0.75** (0.72–0.77)
School policy awareness		0.69 (0.66–0.72)	0.97 (0.91–1.04)		
School bonding		0.77 (0.75–0.79)	0.96 (0.92–0.99)		**0.96** (0.92–0.99)
School Membership		0.55 (0.53–0.58)	0.85 (0.78–0.92)		**0.84** (0.79–0.89)
Perception of peer drug use in school		1.48 (1.44–1.52)	1.11 (1.07–1.15)		**1.13** (1.08–1.17)
**Family Domain**					
Parental monitoring		0.94 (0.94–0.94)	0.96 (0.95–0.96)		**0.96** (0.95–0.96)
Maltreatment		1.96 (1.88–2.05)	1.47 (1.39–1.56)		**1.49** (1.41–1.58)
**Community Domain**					
Community disorganization		1.52 (1.48–1.56)	1.21 (1.17–1.26)		**1.21** (1.17–1.26)
**SCHOOL LEVEL**					
**School Type**					
Municipal state-funded		1		1	1
Subsidized		0.85 (0.77–0.93)		0.86 (0.78–0.95)	0.98 (0.89–1.07)
Private		0.64 (0.51–0.81)		0.61 (0.49–0.77)	1.16 (0.93–1.45)
**Number of Teachers Per School**					
Small		1		1	1
Medium		1.39 (1.26–1.54)		1.41 (1.28–1.55)	0.95 (0.86–1.06)
Large		1.29 (1.08–1.54)		1.25 (1.05–1.48)	**0.75** (0.63–0.90)
**School Denomination**					
Secular		1		1	
Catholic		0.89 (0.81–0.99)		0.95 (0.85–1.06)	
Religious non-Catholic		0.82 (0.68–1.00)		0.86 (0.71–1.04)	
**School Co-Educational Status**					
Only boys		1			
Only girls		1.36 (0.95–1.93)			
Mixed		1.17 (0.87–1.58)			
**Random Intercept**					
Beta	0.27		0.39	0.48	0.39
ICC (%)	7.5		4.4	6.6	4.4
C-statistic (95%CI)					0.77 (0.77–0.78)

*ICC, Intra-Class Correlation. Empty cells indicate that the variables did not enter into the model. Model 0, Null model; Model 1, Univariable association; Model 2, Multivariable associations, only Individual-level variables; Model 3, Multivariable associations, only school-level variables; Full model, Multivariable associations, Individual-level and school-level variables. Significant odds ratios (ORs) are shown in bold (p ≤ 0.05) only for the Full model*.

At the individual level, the older the student, the higher the risk for smoking. Females had also a higher risk (OR = 1.50; 95%CI: 1.40–1.61), independent of whether they attended a mixed or only-girls school. In the school domain, higher academic achievement (OR = 0.75; 95%CI: 0.72–0.77), stronger sense of school membership (OR = 0.84; 95%CI: 0.79–0.89), and a good sense of school bonding (OR = 0.96; 95%CI: 0.92–0.99) reduced the risk for smoking. On the contrary, students who perceived more using, passing, or selling drugs at school had a higher risk. In the family domain, parental monitoring seemed to reduce the risk for smoking (OR = 0.96; 95%CI: 0.95–0.96). However, reporting a higher frequency of maltreatment actions at home increased the risks (OR = 1.49; 95%CI: 1.41–1.58). The perception of actions of violence and community disorganization also increased the risk for smoking.

At the school level, the only factor associated with smoking was the number of teachers per school. The higher the number, the lower the risk for smoking (OR = 0.75; 95%CI: 0.63–0.90).

### Drinking alcohol and associated factors

The full model had a good fit (C-statistic = 0.78). Regarding bullying experience, students who had any kind of bullying experience, compared with students with no bullying experience, had a higher risk for drinking; and the risk was highest among those who bullied others. The risks were similar between victims (OR = 1.23; 95%CI: 1.10–1.38) and bystanders (OR = 1.26; 95%CI: 1.14–1.38) and between bullies (OR = 1.81; 95%CI: 1.55–2.13) and bully-victims (OR = 1.72; 95%CI: 1.58–2.03) (see Table 5).

**Table 5:** Multilevel logistic regression analysis: alcohol use.

		**Alcohol use**			
	**Model 0**	**Model 1**	**Model 2**	**Model 3**	**Full model**
		**OR (95%CI)**	**OR (95%CI)**	**OR (95%CI)**	**OR (95%CI)**
**INDIVIDUAL LEVEL**					
**Personal Domain**					
Age		1.49 (1.47–1.51)	1.50 (1.47–1.53)		**1.50** (1.47–1.53)
Gender (ref. Male)		1.06 (1.01–1.12)	1.25 (1.17–1.34)		**1.23** (1.15–1.32)
**Bullying Experience**					
No experience		1	1		1
Bystander only		1.53 (1.41–1.66)	1.23 (1.11–1.36)		**1.26** (1.14–1.38)
Victim only		1.57 (1.42–1.72)	1.19 (1.06–1.34)		**1.23** (1.10–1.38)
Bully only		2.72 (2.38–3.11)	1.75 (1.48–2.06)		**1.81** (1.55–2.13)
Bully-victim only		2.72 (2.46–3.01)	1.76 (1.54–2.00)		**1.72** (1.58–2.03)
**School Domain**					
GPA		0.79 (0.78–0.81)	0.91 (0.88–0.94)		**0.90** (0.87–0.92)
School policy awareness		0.67 (0.65–0.70)	0.97 (0.91–1.04)		
School bonding		0.80 (0.79–0.82)	1.00 (0.96–1.03)		
School membership		0.56 (0.54–0.58)	0.80 (0.74–0.87)		**0.80** (0.76–0.84)
Perception of peer drug use in school		1.43 (1.39–1.47)	1.02 (0.98–1.07)		
**Family Domain**					
Parental monitoring		0.94 (0.94–0.95)	0.96 (0.95–0.96)		**0.96** (0.95–0.96)
Maltreatment		1.86 (1.79–1.94)	1.51 (1.43–1.60)		**1.51** (1.43–1.60)
**Community Domain**					
Community disorganization		1.48 (1.44–1.53)	1.20 (1.16–1.25)		**1.21** (1.16–1.25)
**SCHOOL LEVEL**					
**School Type**					
Municipal state-funded		1		1	1
Subsidized		1.13 (1.02–1.24)		1.15 (1.05–1.27)	**1.25** (1.13–1.39)
Private		1.52 (1.22–1.91)		1.43 (1.16–1.77)	**2.37** (1.90–2.96)
**Number of Teachers Per School**					
Small		1		1	1
Medium		1.64 (1.49–1.80)		1.61 (1.47–1.77)	1.01 (0.91–1.12)
Large		1.55 (1.31–1.84)		1.55 (1.31–1.83)	0.90 (0.76–1.08)
**School Denomination**					
Secular		1		1	1
Catholic		1.02 (0.92–1.13)		0.94 (0.84–1.04)	1.06 (0.95–1.19)
Religious non-Catholic		0.76 (0.62–0.92)		0.77 (0.64–0.94)	**0.71** (0.58–0.86)
**School Co-Educational Status**					
Only boys		1			
Only girls		1.19 (0.84–1.69)			
Mixed		0.97 (0.73–1.30)			
**Random Intercept**					
Beta	0.54		0.51	0.48	0.45
ICC (%)	8.1		7.2	6.6	5.8
C-statistic (95%CI)					0.78 (0.77–0.78)

*ICC, Intra-Class Correlation. Empty cells indicate that the variables did not enter into the model. Model 0, Null model; Model 1, Univariable association; Model 2, Multivariable associations, only Individual-level variables; Model 3, Multivariable associations, only school-level variables; Full model, Multivariable associations, Individual-level and school-level variables. Significant odds ratios (ORs) are shown in bold (p ≤ 0.05) only for the Full model*.

At the individual level, other associated factors were higher age and being female (OR = 1.23; 95%CI: 1.15–1.32). Those students who had good academic performance (OR = 0.90; 95%CI: 0.87–0.92) and had a high sense of school membership (OR = 0.80; 95%CI: 0.76–0.84) had a reduced risk for drinking. Parental monitoring also reduced the risk of drinking (OR = 0.96; 95%CI: 0.95–0.96). On the contrary, students who reported higher acts of maltreatment at home (OR = 1.51; 95%CI: 1.43–1.60) and/or a higher perception of violent or crime behavior near their homes (OR = 1.21; 95%CI: 1.16–1.25), demonstrated an increased risk of drinking.

At the school level, subsidized (OR = 1.25; 95%CI: 1.13–1.39) and private (OR = 2.37; 95%CI: 1.90–2.96) schools had a higher risk for drinking. Finally, students attending religious, non-Catholic schools had a reduced risk for drinking.

### Cannabis use and associated factors

The full model had a good fit (C-statistic = 0.81). Regarding the experience of bullying, those students who participated as bystanders (OR = 1.30; 95%CI: 1.14–1.48), bullies (OR = 2.05; 95%CI: 1.69–2.48), and bully-victims (OR = 1.59; 95%CI: 1.35–1.87) had an increased risk for cannabis use. Victims had a higher risk, but not at a significant level (see Table 6).

**Table 6:** Multilevel logistic regression analysis: cannabis use.

	**Cannabis use**				
	**Model 0**	**Model 1**	**Model 2**	**Model 3**	**Full model**
		**OR (95%CI)**	**OR (95%CI)**	**OR (95%CI)**	**OR (95%CI)**
**INDIVIDUAL LEVEL**					
**Personal Domain**					
Age		1.44 (1.41–1.46)	1.41 (1.37–1.44)		**1.41** (1.37–1.44)
Gender (ref. Male)		0.89 (0.83–0.95)	1.06 (0.98–1.15)		1.06 (0.97–1.15)
**Bullying Experience**					
No experience		1	1		1
Bystander only		1.71 (1.53–1.91)	1.27 (1.11–1.45)		**1.30** (1.14–1.48)
Victim only		1.83 (1.62–2.06)	1.09 (0.94–1.27)		1.11 (0.95–1.29)
Bully only		3.63 (3.09–4.25)	1.96 (1.61–2.38)		**2.05** (1.69–2.48)
Bully-victim only		3.31 (2.92–3.76)	1.56 (1.33–1.84)		**1.59** (1.35–1.87)
**School Domain**					
GPA		0.72 (0.70–0.74)	0.83 (0.80–0.86)		**0.83** (0.80–0.86)
School policy awareness		0.60 (0.57–0.62)	0.93 (0.87–1.01)		
School bonding		0.74 (0.72–0.76)	0.97 (0.93–1.01)		
School Membership		0.47 (0.45–0.49)	0.78 (0.71–0.86)		**0.74** (0.69–0.79)
Perception of peer drug use in school		1.69 (1.64–1.75)	1.22 (1.16–1.27)		**1.23** (1.18–1.29)
**Family Domain**					
Parental monitoring		0.93 (0.93–0.94)	0.96 (0.95–0.96)		**0.96** (0.95–0.96)
Maltreatment		1.97 (1.89–2.06)	1.47 (1.38–1.56)		**1.47** (1.38–1.57)
**Community Domain**					
Community disorganization		1.73 (1.68–1.78)	1.37 (1.31–1.42)		**1.36** (1.31–1.42)
**SCHOOL LEVEL**					
**School Type**					
Municipal state-funded		1		1	1
Subsidized		0.95 (0.85–1.07)		0.95 (0.85–1.06)	**1.19** (1.06–1.33)
Private		0.73 (0.55–0.98)		0.69 (0.52–0.92)	**1.35** (1.03–1.77)
**Number of Teachers Per School**					
Small		1		1	1
Medium		1.53 (1.36–1.73)		1.55 (1.37–1.74)	0.96 (0.85–1.08)
Large		1.68 (1.36–2.07)		1.67 (1.36–2.06)	0.86 (0.70–1.06)
**School Denomination**					
Secular		1			
Catholic		0.88 (0.78–1.00)			
Religious non-Catholic		0.85 (0.67–1.07)			
**School Co-Educational Status**					
Only boys		1			
Only girls		0.89 (0.58–1.36)			
Mixed		0.85 (0.59–1.20)			
**Random Intercept**					
Beta	0.65		0.49	0.61	0.48
ICC (%)	11.4		6.9	10.3	6.6
C-statistic (95%CI)					0.81 (0.81–0.82)

*ICC, Intra-Class Correlation. Empty cells indicate that the variables did not enter into the model. Model 0, Null model; Model 1, Univariable association; Model 2, Multivariable associations, only Individual-level variables; Model 3, Multivariable associations, only school-level variables; Full model, Multivariable associations, Individual-level and school-level variables. Significant odds ratios (ORs) are shown in bold (p ≤ 0.05) only for the Full model*.

At the individual level, age was a strong associated factor. Additionally, those students who performed well the previous year (OR = 0.83; 95%CI: 0.80–0.86), and those who had a strong sense of school membership (OR = 0.74; 95%CI: 0.69–0.79) displayed a lower risk. Parental monitoring was also a strong variable associated with a reduced risk for cannabis use (OR = 0.96; 95%CI: 0.95–0.96). Conversely, those students who reported maltreatment at home had a higher risk (OR = 1.47; 95%CI: 1.38–1.57). Finally, the higher the perception of crime and drug use in the community, the higher the risk for cannabis use (OR = 1.36; 95%CI: 1.31–1.42).

At the school level, students attending subsidized (OR = 1.19; 95%CI: 1.06–1.33) and private (OR = 1.35; 95%CI: 1.03–1.77) schools appeared to have a higher risk for cannabis use than those students attending state schools.

## Discussion

We present the results of one of the largest studies in Latin America exploring the association between the experience of bullying actions and substance use, among a representative sample of adolescents in Chile. We could control this association for several individual-level and school-level variables, using multilevel, multivariable logistic regression models.

Firstly, we found that there is significant inter-school variability for each substance use explored, where cannabis use was the behavior with the highest variance explained by schools (11.4%). After controlling for several variables (seen in the final models), the variance explained by school was reduced. However, there was still unexplained variance, which could be due to other unmeasured variables, such as teaching quality, quality of management by school authorities, the existence of preventive programs, and enforcement of norms. When we compared the contribution to the school effect between individual- and school-level variables, most of the reduction of the inter-school variability is due to pupil composition (individual-level variables), rather than school variables.

We have found that most students (85%) had played some part in bullying dynamics. As it has been found elsewhere, victimization was higher than being a bully (Menesini and Salmivalli, 2017). Furthermore, we found a higher prevalence of substance use, compared with other developed countries (Johnston et al., 2016).

We could say that students who participated in the cycle of bullying (victim, bullies, and bystanders) had a higher risk for substance use. There is evidence that bullies, especially those who are also victims (bully-victims), are in a higher risk for cigarette, alcohol, and cannabis use, which confirms results found in other studies (Kaltiala-Heino et al., 2000; Helstrom et al., 2004; Carlyle and Steinman, 2007; Niemelä et al., 2011). One explanation for this relationship may be that adolescents who display violence and aggressive behaviors to others, may increase their involvement with other adolescents with deviant behaviors, such as substance use (Brook and Newcomb, 1995; Durand et al., 2013). On the other hand, individuals that were victims only were also more at risk, but mainly for cigarettes and alcohol.

The evidence regarding victims is less clear (Durand et al., 2013). For instance, some authors have found that victims are less likely to engage in cigarette smoking (Liang et al., 2007), alcohol, and other drugs (Houbre et al., 2006) than students not involved in bullying. However, others have found similar results to ours, that is, victims of bullying displaying a higher risk for cigarette and alcohol use (Niemelä et al., 2011; Radliff et al., 2012). Students who are victims, may use cigarettes to reduce the anxiety produced by the constant aggression or use substances to increase their social image among their peers (Durand et al., 2013). However, much more research is needed to fully understand why victims use substances.

Regarding bystanders, this is one of the few studies exploring this population. We found a higher risk in this group for alcohol and tobacco use, of an equivalent magnitude to that found among victims. However, we also found that this population had a higher risk for cannabis use, even though at a lower degree than the risk found for bullies. Studying the relationship between observing bullying at school and substance use is in its infancy (Durand et al., 2013). As such, few other studies have explored this association directly (Rivers et al., 2009). As a group, bystanders seem to be more alike to victims; therefore, the potential explanation of the association may be similar. These students may experience psychological co-victimization through their empathic understanding of the suffering of the victims (D'augelli et al., 2002; Durand et al., 2013), may feel anxious at the possibility of being victimized at some point in the future (Durand et al., 2013), or remembering experiences from the past. There is also important evidence of the psychological distress suffered by children and adolescents exposed to violence (Kitzmann et al., 2003). We may also consider it important to explore other outcomes related to students that are bystanders. For example, to study if being exposed to bullying actions is associated with other risk behaviors, such as early sexuality, adolescent pregnancy, suicide, and so on. A recent study found that feelings of helplessness, followed by frequency of observed bullying perpetration, was associated with suicide ideation (Rivers and Noret, 2013). Further research is needed to understand this population better.

We have found that the association between being involved in bullying and substance use seems to be independent from having been exposed to violence in other contexts (e.g., in the community) or having suffered domestic violence. Maltreatment at home had a robust influence on smoking, drinking or cannabis use in our study. The same is true for community disorganization which included the perception of violence in the neighborhood. Similar findings have been found elsewhere (Wright et al., 2013). Further research should be done to investigate the mechanisms involved in these associations, because exposure to different types of violence (bullying, domestic, and community violence) may have different mediating factors.

Parental monitoring appears to reduce the likelihood of substance use. We have confirmed here our previous results regarding the influence of parental monitoring (Gaete and Araya, 2017), using a different nationally representative sample. Future preventive programs in Chile may need to consider these findings to find ways of empowering parents with information and training on strategies to set clearer rules and exert effective control. Finally, we also found that students who had a stronger sense of school membership are less likely to use substances. Recent reports have provided evidence that school-based interventions aiming to increase school climate and school membership may improve different students' outcomes including substance use (Bonell et al., 2014; Langford et al., 2014, 2015). Therefore, future preventive interventions may also need to consider actions aiming to increase the psychological sense of school belonging among students.

### Limitations

Among the limitations, the significant associations identified cannot be considered as causal relationships, as the current research used a cross-sectional study design. Additionally, self-report of bullying experience may be affected by some bias, such as for instance reporting bias as some students may be reluctant to report bullying to others. Even though it is expensive, direct observations in schools may help to clarify differences in the prevalence of victimization and perpetration. We also cannot consider the bystander population as a homogeneous group. From the literature reviewed, we may identify at least four different types of witnesses (National Academies of Sciences, Engeering, and Medicine, 2016): two types may support bullies (one may help the perpetrator once the bullying starts, and other may encourage bullies by showing signs of approval); others may defend victims against bullying; and a final role for neutral students (i.e., those who are there, but support neither the victim nor the bully). Therefore, it is important to be more specific when studying the associating between observing bullying and substance use. Something similar may also be said regarding victims and bullies, who are not homogeneous groups either.

Future research in low and middle-income countries need to address these limitations. Furthermore, there is some evidence to support the idea that preventing one behavior (bullying) may have a positive effect on another behavior, such as decreasing substance use. Of interest is the association found between a stronger sense of school membership and a reduced risk for substance use. This variable seems to have impacts on several other domains, such as for instance, academic performance.

## Conclusions

This study shows that bullies and bully-victims have a higher risk for cigarette, alcohol, and cannabis use. Individuals that were victims also demonstrated a high risk for cigarette and alcohol use; and bystanders displayed a high risk for cigarette, alcohol, and cannabis use. This is one of the few studies exploring the association between witnesses of bullying and substance use. These findings add new insights to the study of the co-occurrence of bullying and substance use. However, the mechanisms involved in these associations remain to be elucidated. We have provided good evidence that bystanders are an at-risk group, and this information should be taken into account when designing preventive interventions. Additionally, we have identified other factors related to substance use, such as school membership and parental monitoring, which can be considered in preventive interventions. Future research may also consider the potential benefits of implementing a preventive intervention to address one of these problems (e.g., bullying) and to study the effect on other problems (e.g., substance use).

## Author contributions

JG conceived the study, performed the statistical analyses, and drafted the manuscript. BT, DV, CR, CS, and RA participated in the interpretation of the data and contributed to the writing. EV participated in the design and coordination of the study, data collection and contributed to the writing. All authors read and approved the final manuscript.

### Conflict of interest statement

The authors declare that the research was conducted in the absence of any commercial or financial relationships that could be construed as a potential conflict of interest.
